# Serum Folate Levels and Lung Cancer Risk: A Meta-Epidemiological Study of Population-based Case-Control Studies

**DOI:** 10.31557/APJCP.2020.21.6.1829

**Published:** 2020-06

**Authors:** Jong-Myon Bae

**Affiliations:** *Department of Preventive Medicine, Jeju National University College of Medicine, Jeju, Republic of Korea.*

**Keywords:** Folic acid, lung neoplasm, systematic review, meta-analysis

## Abstract

**Objective:**

While it has been claimed that lung cancer occurs due to epigenetic mechanisms, four systematic reviews were reported to investigate the association between serum folate levels and lung cancer risk. Considering some methodological problems founded in the systematic review, a meta-epidemiological study was conducted.

**Methods:**

The selection criteria of this study were defined that a case-control study was conducted to determine the risk of lung cancer occurrence according to the concentration of serum folate and its results showed odds ratio and its 95% confidence interval. Additional paper was explored from cited lists of 4 papers selected by previous systematic reviews. Random effect model was applied if I-squared value was over 50%.

**Results:**

For 5 case-control studies selected, the summary odds ratios (and their 95% confidence intervals) were 0.82 (0.74-0.90) in men, 0.70 (0.62-0.79) in former smokers, and 0.86 (0.75-1.00) in non-smokers.

**Conclusion:**

Higher foliate levels can decrease lung cancer risk in men and former smokers. Especially, the protective effect was highest in former smokers compared in non-smokers and current smokers. Based on these facts, folate fortification programs to reduce lung cancer risk would be focused on former smokers in men. And some epidemiological studies are needed to provide a hypothesis to explain the sex differences in the association between folate and lung cancer risk.

## Introduction

Lung cancer ranks first in incidence and mortality rates worldwide (Bray et al., 2018; Barta et al., 2019), and smoking is the best-known risk factor (Bae et al., 2013; Barta et al., 2019). Recently, it has been claimed that lung cancer occurs due to epigenetic mechanisms caused by exposure to air pollution (Vaid and Floros, 2009; Wen et al., 2011; Bae, 2018a).

Epigenetic alteration means that genetic expression changes occur while DNA information remains intact (Bae, 2018b), and refers to DNA methylation, histone modification, small RNA, and so on in the aspect of cellular molecule (Blair and Yan, 2012). While folate (vitamin B_9_) is known to be involved in the DNA methylation process (Blount et al., 1997), 4 systematic reviews were reported to investigate the association between serum folate levels and lung cancer risk (Takata et al., 2012; Dai et al., 2013; Zhang et al., 2015; Yang et al., 2018) (Table 1). Two systematic reviews presented meta-analysis results with odds ratio reported no statistical significance (Takata et al., 2012; Dai et al., 2013), but another 2 systematic reviews presented by standardized mean differences showed statistically significant differences (Zhang et al., 2015; Yang et al., 2018). The reasons for the different results among systematic reviews would be as follows. First, the selection criteria for meta-analysis have changed due to the difference of summary index. Because Johansson et al., (2010) was excluded from the 2 systematic reviews presented by standardized mean differences. Second, it seems not to strict in applying the proposed selection criteria. In the most recently reported Yang et al., (2018), the heterogeneity was very high (I-squared value =89.4%), 6 of the 14 papers selected had 5 or less point on the Newcastle-Ottawa Scale, and 10 papers did not provide smoking history information.

Accordingly, it is necessary to conduct another meta-analysis using odds ratio with applying more stringent selection criteria. In order to re-investigate the association between serum folate levels and the lung cancer risk, a meta-epidemiological study was conducted (Bae, 2014). 

## Materials and Methods

As the main aim of meta-epidemiological study is to evaluate problems associated with errors that can occur while performing a systematic review (Bae, 2014), the subjects of analysis in meta-epidemiological study are original articles selected in systematic reviews (Murad and Wang, 2017). Considering previous systematic reviews’ problems pointed out in introduction, the selection criteria of this meta-epidemiological study were as follows. A case-control study was conducted to determine the risk of lung cancer occurrence according to the concentration of serum folate and its results showed odds ratio and its 95% confidence interval (95% CI). 

Applying the selection criteria, a total of four case-control studies (Hartman et al., 2001; Johansson et al., 2010; Durda et al., 2017; Fanidi et al., 2018) were selected among the papers selected from the 4 systematic reviews presented in Table 1. For each of these 4 case-control studies and 4 systematic reviews, a search list was created using “cited by” option of citation discovery tools suggested by PubMed (Bae and Kim, 2016). In the search list, potential papers were explored by applying the above selection criteria.

In each of the finally selected papers, the information to be used for meta-analysis was extracted in two ways. The odds ratio of the highest category and its 95% CI value were taken as information extracted by the highest versus lowest method. And the interval collapsing method was applied to utilize the information of all categories (Bae, 2016). In other words, a meta-analysis of the results of the other categories except for the reference category was carried out, and then the summary odds ratio was counted as information extracted by the interval collapsing method of the paper. Logarithm odds ratio and its standard error were calculated from the extracted information.

The heterogeneity level was evaluated by the I-squared value (%), and the meta-analysis was applied to the random effect model for 50% or more of I-squared value (Harris et al., 2008). Subgroup analyses were performed according to the sex – men and women – and smoking habits – non smokers, former smokers, and current smokers. Egger test was conducted to check for publication bias (Sedgwik, 2015). Statistical significance level was calculated as 0.05.

## Results

As of December 31, 2019, a total of 90 papers cited 8 papers (Hartman et al., 2001; Johansson et al., 2010; Takata et al., 2012; Dai et al., 2013; Zhang et al., 2015; Durda et al., 2017; Fanidi et al., 2018; Yang et al., 2018). When the selection criteria were applied, one case-control study published in 2019 could be added (Stanisławska-Sachadyn et al., 2019). Therefore, 5 case-control studies were finally selected for meta-analysis (Hartman et al., 2001; Johansson et al., 2010; Durda et al., 2017; Fanidi et al., 2018; Stanisławska-Sachadyn et al., 2019). 

Table 2 lists values of the highest versus lowest method and the interval collapsing method values for 13 databases (a-m), classified by sex and smoking habits from the 5 selected case-control studies. The summary odds ratio value for five databases (a, b, f, g, m), which are the results of all subjects, was 0.87 for both the highest versus lowest method and the interval collapsing method and included 1 in 95%CI (Table 3). Egger’s test showed no publication bias from them (P=0.826).

Table 3 shows the summary effect sizes of subgroup analyses by sex and smoking habits with applying the extracted values from Table 2. The summary odds ratio using values of the interval collapsing method were 0.82 (95%CI: 0.74-0.90) in men and 0.70 (95%CI: 0.62-0.79) in former smokers and showed statistically significant. In non smokers, the summary odds ratio using value of the interval collapsing method was 0.86 (95%CI: 0.75-1.00) and showed a marginally statistical significance (P=0.052).

Table 4 shows the results of sensitivity analysis to determine the effect of Stanisławska-Sachadyn et al. (2019) added by searching using citation discovery tools. This is because the odds ratio in Stanisławska-Sachadyn et al. (2019) (‘m’ database in Table 2) is the only one whose odds ratio direction is greater than 1. When the ‘m’ database was excluded, all subjects had a statistical significance, but current smokers had still no statistical significance while the I-squared value decreased from 81.0% into 52.7%.

**Table 1:** Results of Systemaatic Reviews to Evaluate the Association between Serum Folate Level and Lung Cancer Risk

FA (PY)	Takata (2012)	Dai (2013)	Zhang (2015)	Yang (2018)
Search to	NA	Feb 2013	Nov 2013	Feb 2018
Selected	4	4	5	14
I-squared value	<0.01	0.06	NA	89.4
Summary ES	OR=0.76	OR= 0.77	SMD= -1.91	SMD= -0.53
(95% CI)	(0.58, 1.00)	(0.59, 1.01)	(-3.04, -0.78)	(-0.70, -0.35)

CI, confidence interval; ES, effect size; FA, first author; NA, not available; OR, odds ratio; PY, publication year, SMD, standardized mean differences

**Table 2:** Results of 13 Databases by Sex and Smoking Habits

FA	PY	Sex	Smoking	OR (95% CI) by HLM	OR (95% CI) by ICM	Database
Hartman	2001	M	A	0.96 (0.52-1.79)	0.78 (0.49-1.26)	a
Johansson	2010	B	A	0.69 (0.50-0.95)	0.79 (0.66-0.94)	b
		B	N	0.84 (0.43-1.65)	0.98 (0.67-1.43)	c
		B	F	0.58 (0.37-0.91)	0.67 (0.52-0.86)	d
		B	C	0.54 (0.34-0.83)	0.77 (0.62-0.98)	e
Durda	2017	B	A	0.60 (0.38-0.94)	0.74 (0.57-0.97)	f
Fanidi	2018	B	A	0.86 (0.74-0.99)	0.87 (0.81-0.94)	g
		M	A	0.75 (0.61-0.93)	0.82 (0.74-0.90)	h
		W	A	0.97 (0.79-1.19)	0.94 (0.84-1.05)	i
		B	N	0.86 (0.63-1.17)	0.85 (0.72-0.99)	j
		B	F	0.66 (0.51-0.85)	0.71 (0.62-0.82)	k
		B	C	0.97 (0.77-1.21)	0.93 (0.84-1.03)	l
Stanisławska-Sachadyn	2019	B	C	1.54 (1.04-2.29)	1.54 (1.04-2.29)	m

CI, confidence intervals; FA, first author; HLM, the highest versus lowest method; ICM, the interval collapsing method; NA, non available; OR, odds ratio; PY, publication year; Sex, M (men); W(women); B(both); Smoking, A (adjusted); C (current smokers); F (former smokers); N (non smokers)

**Table 3:** Results of Subgroup Analyses Using Databases in Table 2

Subgroup	Database in Table 2	sOR (95% CI) [I^2] by HLM	sOR (95% CI) [I^2] by ICM
All	a,b,f,g,m	0.87 (0.66-1.15) [68.6]	0.87 (0.74-1.02) [63.1]
Men	a,h	0.77 (0.63-0.94) [0.0]	0.82 (0.74-0.90) [0.0]
Women	i	0.97 (0.79-1.19)	0.94 (0.84-1.05)
Never smoking	c,j	0.86 (0.65-1.14) [0.0]	0.86 (0.75-1.00) [0.0]
Former smoking	d,k	0.77 (0.53-1.12) [55.2]	0.70 (0.62-0.79) [0.0]
Current smoking	e,l,m	0.94 (0.58-1.54) [83.2]	0.98 (0.75-1.29) [77.0]

CI, confidence interval; HLM, highest versus lowest method; I^2, I-squared value; ICM, interval collapsing method; sOR, summary odds ratio

**Table 4:** Results of Sensitivity Analysis for ‘m’ Database (DB) in Table 2

	Database in Table 2	sOR (95% CI) [I^2] by HLM	sOR (95% CI) [I^2] by ICM
All	a,b,f,g,m	0.87 (0.66-1.15) [68.6]	0.87 (0.74-1.02) [63.1]
All excluding ‘m’ DB	a,b,f,g	0.81 (0.72-0.92) [16.3]	0.85 (0.80-0.90) [0.0]
Current smoking	e,l,m	0.94 (0.58-1.54) [83.2]	0.98 (0.75-1.29) [77.0]
Current smoking excluding ‘m’ DB	e,l	0.75 (0.42-1.32) [81.0]	0.88 (0.74-1.04) [52.7]

CI, confidence interval; HLM, highest versus lowest method; I^2, I-squared value; ICM, interval collapsing method; sOR, summary odds ratio

## Discussion

The results of the study showed that the higher the serum folate level in men, former smokers, and non smokers decreased the risk of lung cancer occurrence. The summary odds ratios and their 95%CI of subgroup analyses were as same as Yang et al., (2018) except for current smokers. Although this study applied more stringent selection criteria than Yang et al., (2018), it is assumed that the database applied to the analysis of subgroups by sex and smoking habit was the same. 

Of the 14 papers selected by Yang et al., (2018), 3 papers (Johansson et al., 2010; Durda et al., 2017; Fanidi et al., 2018) met the selection criteria in this study, and their Newcastle-Ottawa Scale was 8 or more. The lower I-squared value from 89.4% to 68.6% could be deduced by applying more stringent selection criteria. From these findings, it can be reaffirmed that suggestion of valid selection criteria in the planning process and the strict application in the evaluation process are particularly important issues in conducting a systematic review.

In a systematic review of nutritional epidemiology studies that present disease risks according to their distribution in a particular food or nutrient, the highest versus lowest method has a limit to ignore some information (Bae, 2016). In both the results of each paper (Table 2) as well as the results of meta-analysis (Tables 3 and 4), the interval collapsing method showed that the confidence interval was narrowed, and the heterogeneity decreased compared to the highest versus lowest method. Especially, in the former smokers of Table 3, the highest versus lowest method showed no statistical significance with 55.2% of I-squared value, but the interval collapsing method took a statistical significance with 0% of I-squared value after narrowing the confidence interval of summary odds ratio. Considering these facts, the interval collapsing method has another evidence for reducing the heterogeneity between articles with using the all information presented by each of the selected papers.

The level of folate in nutritional epidemiology is determined by measuring serum and RBC concentrations, using the level of intake through questionnaires, or using the dose administered during trials (Brasky et al., 2017; Stanisławska-Sachadyn et al., 2019). Since the serum concentration reflects a recent uptake (Duthie, 2011), this study selected case-control studies that measured serum folate. Interestingly, systematic reviews of prospective cohort studies reported the same result that there was no link between intake of folate and lung cancer risk (Cho et al., 2006; Zhang et al., 2014). In particular, the subgroup analysis of dose-response meta-analysis (Zhang et al., 2014) showed the same result that the protective effect was found in men. In other words, different measures of serum concentrations and intake levels for folate were applied, but the same conclusion was found to be protective in men.

Sex differences in the association between folate and lung cancer could be considered as follows. First, to date, there is only one database that examined the incidence of female lung cancer according to serum foliate levels (Table 2). But, Cho et al., (2006) reported the pooled relative risk of lung cancer according to folate intake levels as 0.86 (0.54-1.38) in men and 1.12 (0.93-1.34) in women from the 8 prospective cohorts. It is difficult to explain the sex difference due to the lack of research data in that the direction of risk by sex was different even though there was no statistical significance. Second, consider the social notion that there are more smokers in men than women. However, there were no associations in current smokers more with men, while the former and non smokers showed statistical significance (Table 3). Furthermore, a cohort of nonsmoking women reported no relationship between folate intake levels and lung cancer incidences (Takata et al., 2012). Some epidemiological studies are needed to provide a hypothesis to explain the sex differences in the association between folate and lung cancer risk. Considering the histological differences of lung cancer between men and women (Barta et al., 2019), additional epidemiologic studies are needed to evaluate the association by histological type, not overall lung cancer. Last, there have been reports that high levels of folate might promote cancers (Kim, 2004; Ulrich and Potter, 2006; Dai et al., 2013). In the results of the dose-response meta-analysis (Zhang et al., 2014), as the daily intake increases, the men’s summary odds ratio were 0.85 (0.69-1.06), 0.77 (0.53-1.11), and 077 (0.66-0.89), but women’s summary odds ratio were 0.78 (0.63-0.97), 0.86 (0.66-1.13), 1.02 (0.85-1.22). In other words, the higher the folate intake, the stronger the protective effect of lung cancer in men, but the protective effect disappeared in women. Based on the recent report (Stanisławska-Sachadyn et al., 2019) that the sex-specific effects of SLC19A1 c.80G>A polymorphism on lung cancer among men and women were different, further studies of genome epidemiology are needed. 

In conclusions, higher foliate levels can decrease lung cancer risk in men and former smokers. Especially, the protective effect was highest in former smokers compared in non-smokers and current smokers. Based on these facts, folate fortification programs to reduce lung cancer risk would be focused on former smokers in men.
